# Long-term imaging characteristics of the pancreas related to diabetes induced by immune checkpoint inhibitor therapy

**DOI:** 10.1007/s00592-026-02683-8

**Published:** 2026-05-26

**Authors:** Seiji Tomofuji, Shin Urai, Kei Yoshino, Hironori Bando, Yushi Hirota

**Affiliations:** 1 Division of Diabetes and Endocrinology, Department of Internal Medicine, Kobe University Graduate School of Medicine, 7-5-2 Kusunoki-cho, Chuo-ku, Kobe, 650-0017 Japan; 2 Division of Diabetes and Endocrinology, Kobe University Hospital, Kobe, Japan; 3 Division of Diabetes and Endocrinology, Hyogo Prefectural Awaji Medical Center, Sumoto, Japan

**Keywords:** Type 1 diabetes, Immune checkpoint inhibitor, PD-1 antibody, Immune-related adverse event, Magnetic resonance imaging

## Abstract

**Background:**

Immune checkpoint inhibitor (ICI)-induced diabetes is a rare endocrine immune-related adverse event, and long-term pancreatic magnetic resonance imaging (MRI) findings after disease onset have not been well characterized.

**Case presentation:**

An individual developed abrupt hyperglycemia during pembrolizumab therapy for recurrent renal cell carcinoma and subsequently required insulin therapy. Serum C-peptide rapidly declined to below the detection threshold, whereas pancreatic enzyme levels remained within normal limits throughout follow-up. MRI performed 1 day after diagnosis demonstrated diffuse high signal intensity on diffusion-weighted imaging (DWI) and reduced apparent diffusion coefficient (ADC) values throughout the pancreas. Serial imaging over 25 months showed progressive pancreatic atrophy, whereas the DWI/ADC features persisted.

**Conclusion:**

This case provides a new descriptive longitudinal radiological observation of persistent diffusion-related pancreatic MRI findings and progressive pancreatic atrophy after the onset of ICI-induced diabetes.

## Introduction

Although immune checkpoint inhibitors (ICIs)—including antibodies to cytotoxic T lymphocyte antigen–4 (CTLA-4), to programmed cell death–1 (PD-1), and to programmed cell death–ligand 1 (PD-L1)—have shown efficacy for the treatment of various malignancies, these agents have been associated with immune-related adverse events (irAEs) that can affect multiple organ systems, including endocrine glands such as the pancreas as well as pituitary, thyroid, adrenal, and parathyroid glands [1].

Among endocrine irAEs, ICI-induced diabetes is a rare (estimated incidence of < 1%) but clinically important complication [1, 2]. Affected individuals often experience acute symptomatic hyperglycemia or diabetic ketoacidosis due to the abrupt and irreversible loss of endogenous insulin secretion, resembling the clinical profile of conventional type 1 diabetes (T1D). In addition to these clinical features, morphological changes in the pancreas have also been reported after the onset of ICI-induced diabetes. Changes in pancreatic volume and atrophy were documented in an early report of ICI-induced diabetes [3], and a recent comprehensive analysis provided additional characterization of these morphological alterations [4]. However, magnetic resonance imaging (MRI) assessments incorporating diffusion-weighted imaging in ICI-induced diabetes remain limited.

Diffusion-weighted magnetic resonance imaging (DWI), which exploits the Brownian motion of water molecules in tissues, has emerged as a useful tool for lesion characterization and early-stage diagnosis. Although originally developed for the detection of acute cerebral infarction and brain tumors, its application has expanded to include abdominal imaging [5]. In the case of the pancreas, DWI has been applied as a noninvasive technique to evaluate potential changes in conditions such as chronic pancreatitis, autoimmune pancreatitis, and T1D [5, 6]. Measurement of the apparent diffusion coefficient (ADC) derived from DWI allows the differentiation between normal and diseased pancreatic tissues [5]. In individuals with T1D, high signal intensity on DWI combined with reduced ADC values has been observed and discussed in relation to tissue alterations in the pancreatic parenchyma [6]. A similar imaging pattern has also been observed at the onset of ICI-induced diabetes [7]. However, whether these diffusion-related abnormalities persist over the long term remains to be characterized.

Here, we report an individual with ICI-induced diabetes who underwent serial pancreatic MRI, including DWI and ADC mapping, with follow-up to 25 months, presenting the longitudinal course of these diffusion-related imaging features as descriptive radiological observations.

## Case presentation

### Clinical history, onset, and diagnosis of ICI-induced diabetes

The patient underwent laparoscopic partial nephrectomy for clear cell renal cell carcinoma of the left kidney (pT3aN0M0) at 57 years of age. Eight years later, a local recurrence with renal vein invasion was detected on surveillance computed tomography (CT), and open radical nephrectomy was performed. Pathological evaluation confirmed the diagnosis of recurrent disease (pT4N0M0). Postoperative adjuvant therapy with pembrolizumab (antibody to PD-1) was initiated and continued according to the standard dosing intervals.

At 66 years of age, the patient, who was male, with a height of 162.5 cm and body mass of 77.4 kg (body mass index, 29.3 kg/m²), had no history of metabolic disease or regular medication use. There was no family history of diabetes mellitus. Although no HbA_1c_ measurement was available before ICI therapy, random blood glucose concentrations ranging from 75 to 135 mg/dL before and during the early course of treatment suggested the absence of preexisting diabetes. On day 177 after the initiation of ICI therapy, asymptomatic hyperglycemia was incidentally noted, with a random blood glucose level of 464 mg/dL, serum C-peptide level of 2.41 ng/mL (measured by a one-step sandwich enzyme immunoassay using the E-test TOSOH II [C-peptide II] kit [Tosoh Corporation, Tokyo, Japan]; reference range, 0.69–2.45 ng/mL), and HbA_1c_ level of 6.1%. Two days later, the patient presented with polydipsia and polyuria. The random blood glucose level was 684 mg/dL, and the C-peptide level was 1.63 ng/mL. Total ketone bodies were elevated (1598 µmol/L), but gastrointestinal symptoms and metabolic acidosis were not present, with a bicarbonate level of 22.9 mmol/L. The HbA_1c_ level of 6.6% was disproportionately low relative to the hyperglycemia, consistent with rapid disease progression.

The patient tested negative for islet-related autoantibodies—including those to glutamic acid decarboxylase (GAD), insulinoma-associated antigen-2 (IA-2), and zinc transporter 8 (ZnT8)—consistent with previous reports of the absence of detectable autoantibodies in a subset of ICI-induced diabetes cases. On the basis of the abrupt onset of insulin dependence, the lack of autoantibodies, and the clinical presentation fulfilling the established criteria for conventional T1D, a diagnosis of ICI-induced diabetes was made 179 days after ICI initiation. HLA typing showed no T1D risk haplotypes known in Japanese individuals, including DRB1*04:05–DQB1*04:01.

Multiple daily insulin injections were initiated promptly after the diagnosis. On day 180 after ICI initiation (the day after the diagnosis of ICI-induced diabetes), the fasting blood glucose level of the patient was 303 mg/dL and his C-peptide level had decreased to 0.93 ng/mL. Over the subsequent 2 weeks, the C-peptide level dropped further, ultimately falling below the detection threshold (< 0.01 ng/mL), indicating the complete loss of endogenous insulin secretion. From the time of diagnosis onward, serum levels of pancreatic enzymes, including amylase and lipase, remained within normal limits, and no symptoms suggestive of exocrine pancreatic dysfunction were observed. Although specialized tests such as those for fecal elastase-1 were not performed, and it is recognized that fecal elastase-1 levels can be reduced even in asymptomatic individuals with T1D, the absence of abdominal symptoms and the long-term stability of serum enzyme levels did not suggest overt clinical exocrine pancreatic insufficiency; however, subclinical impairment cannot be excluded throughout the clinical course.

Throughout the follow-up period, no glucocorticoid or pancreatotoxic agents were administered, and no other irAEs were detected.

### Longitudinal pancreatic imaging and quantitative ADC analysis

**Fig. 1:**
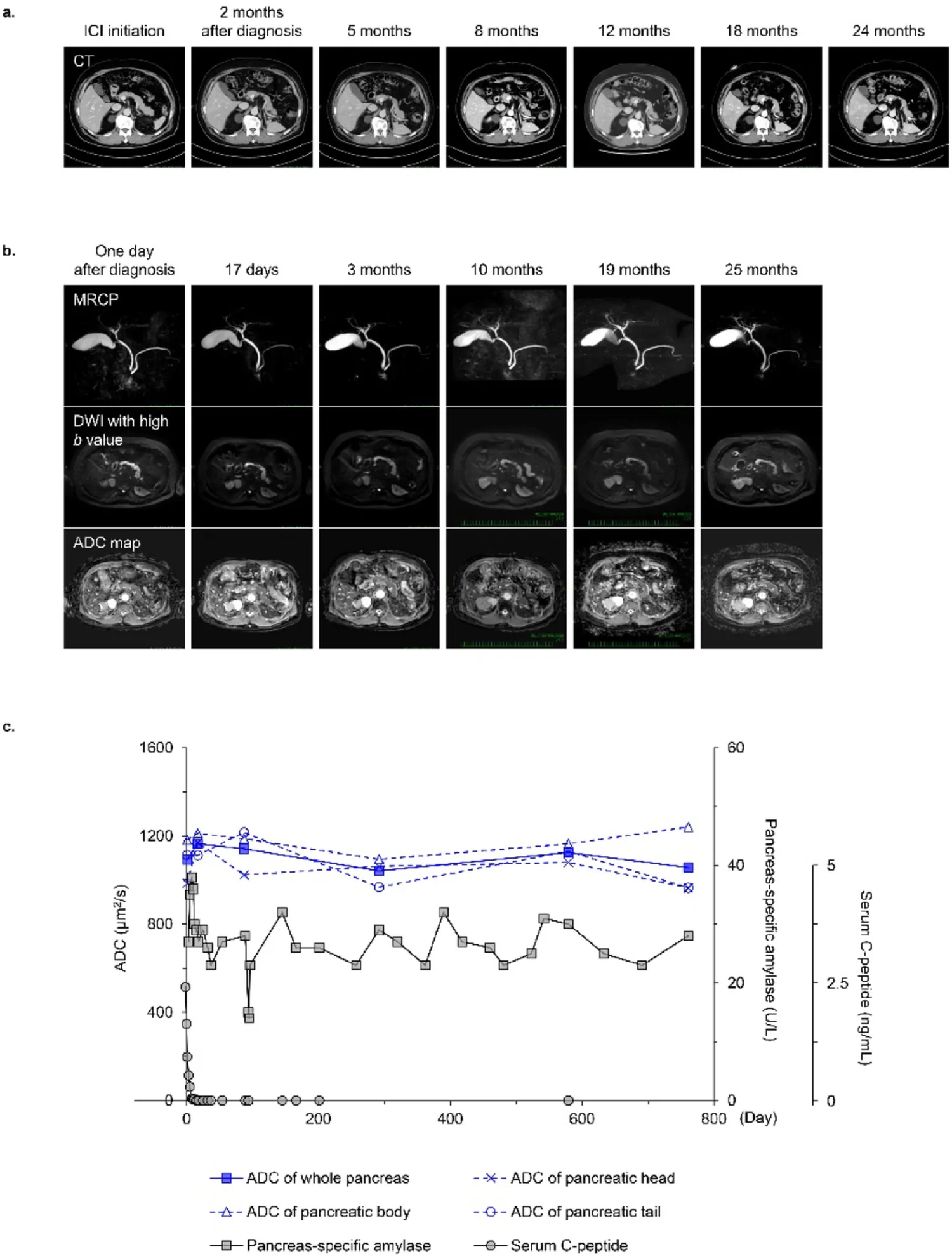
Longitudinal pancreatic imaging and quantitative trajectories. (a) Serial CT scans obtained during follow-up showing pancreatic atrophy over time after the onset of ICI-induced diabetes. (b) Representative MRI (diffusion-weighted imaging [DWI] and apparent diffusion coefficient [ADC] maps) obtained 1 day after diagnosis, demonstrating diffuse high signal intensity on DWI and reduced ADC throughout the pancreas. Similar diffusion-related imaging features were observed on subsequent MRI scans through 25 months of follow-up. (c) Longitudinal trajectories of whole-pancreas and regional (head, body, tail) ADC values (left y-axis) and serum pancreatic amylase and C-peptide levels (right y-axes), including the time point when serum C-peptide fell below the detection threshold (< 0.01 ng/mL)

CT revealed no metastatic disease and no overt morphological abnormality of the pancreas, including no ductal dilatation, cystic lesion, focal mass, or parenchymal enlargement (Fig. 1a). MRI performed 1 day after the diagnosis of ICI-induced diabetes revealed a diffuse high signal intensity on DWI with a reduced ADC throughout the pancreatic parenchyma, supporting a nonneoplastic etiology (Fig. 1b). Although preonset MRI was unavailable, the prior CT findings were unremarkable. These observations, together with normal serological findings and the absence of ductal irregularity or parenchymal enlargement, did not show features typical of autoimmune and chronic pancreatitis.

Following the diagnosis of ICI-induced diabetes, pembrolizumab therapy was discontinued. Serial CT and MRI follow-up was performed as part of routine oncologic surveillance. Surveillance imaging showed no progression until 24 months after the diagnosis of ICI-induced diabetes, when local recurrence was detected on a CT scan. Cabozantinib therapy was subsequently initiated.

A retrospective analysis of the changes in pancreatic imaging findings obtained during the long-term follow-up period was performed. Quantitative ADC values were calculated according to previously described methods [5–7]. Serial MRI was conducted with the same 3 T system (Ingenia 3.0 T, Philips Medical Systems), DWI used two *b* values (0 and 800–1000 s/mm²), and the section thickness was 5 mm. Acquisition parameters were harmonized across serial scans whenever feasible, and region of interest (ROI) placement and measurement procedures were maintained constant to mitigate interscan variability. ROIs were consistently placed within the pancreatic parenchyma across time points, with ducts, vessels, and peripancreatic fat being carefully excluded and each ROI measuring approximately 100 mm². ADC was sampled on three contiguous slices per region (head, body, and tail, when present), and the slice values were averaged to yield the regional and whole-pancreas ADC values. All ROIs were manually delineated by an endocrinologist and independently verified by a second endocrinologist, with discrepancies being resolved by consensus.

Serial CT and MRI scans demonstrated pancreatic atrophy over time (Fig. 1a). The high DWI signal intensity was present throughout 25 months of follow-up, and whole-pancreas ADC values remained consistently low relative to those reported for healthy individuals (on the order of 1600 μm²/s) [5, 6], with similar trajectories across regions (head, body, and tail) (Fig. 1b, c). During this period, glycemic control remained stable (HbA_1c_ of 6.7–8.1%) on a basal-bolus insulin regimen. These diffusion-related imaging features were documented throughout the follow-up, including after serum C-peptide fell below the detection threshold (Fig. 1c).

## Discussion

In this case report, we documented the 25-month longitudinal course of pancreatic diffusion-related MRI features in an individual with ICI-induced diabetes. Throughout the follow-up period, diffuse high signal intensity on DWI and low ADC values were observed, alongside pancreatic atrophy over time and the complete loss of endogenous insulin secretion.

The development of ICI-induced diabetes, similar to that of other endocrine irAEs, has been reported to be associated with improved survival outcomes in a claims-based observational study of ICI-treated individuals [2]. Although this association should be interpreted with caution, the increasing number of survivors after ICI therapy makes the characterization of chronic-phase organ changes of clinical interest. While CT-based assessments have reported pancreatic volume loss and atrophy after the onset of ICI-induced diabetes [1, 3], pancreatic volume and immune biomarkers have also been reported to predict the development of ICI-induced diabetes [4], underscoring the potential for integrating imaging with functional and immunologic profiling. MRI—and particularly DWI/ADC—provides diffusion-related information reflecting water mobility and may complement morphological assessment. Despite this capability, longitudinal DWI/ADC follow-up has rarely been described, with the exception of a single case report confined to acute-phase findings [7]. Accordingly, long-term MRI observations beyond the acute phase remain limited, and the present 25-month follow-up provides a descriptive record of diffusion-related MRI features alongside pancreatic atrophy over time after ICI-induced diabetes onset. However, minor variability in acquisition parameters, including differences in the higher *b* value across scans, should be considered when interpreting absolute ADC values over time.

The biological substrate of the observed MRI signal abnormalities cannot be determined from imaging alone. Diffusion-related abnormalities are non-specific and may reflect multiple processes, including atrophy-related microstructural changes, fibrosis, fat infiltration, perfusion effects, or technical variability. Therefore, the present imaging findings do not allow inference regarding ongoing islet injury or immune activity. Although histopathologic and clinical studies have demonstrated immune-mediated islet injury in ICI-induced diabetes [1], the extent to which such mechanisms contribute to persistent diffusion-related MRI features remains uncertain without direct tissue-based correlation. Notably, these diffusion-related abnormalities were present even after serum C-peptide fell below the detection threshold in this individual, indicating a temporal dissociation between the loss of endogenous insulin secretion and the persistence of radiological features. However, the biological basis of this temporal dissociation remains unknown. Accordingly, this report should be interpreted as a descriptive radiological observation rather than evidence of ongoing pancreatic disease activity.

Several limitations warrant explicit emphasis. First, this is a single-case report without pre-onset MRI, comparator cases (e.g., individuals treated with ICIs who did not develop diabetes, or those with conventional T1D), or histological validation, precluding causal inference and generalization. In addition, formal exocrine function testing (e.g., fecal elastase-1) and assessment of non–beta-cell endocrine components, including alpha-cell glucagon secretion, were not performed; therefore, subclinical exocrine dysfunction or broader islet-cell dysfunction cannot be ruled out, and functional or histological correlates of the diffusion-related MRI features remain undetermined. Second, although serial MRI was performed on the same 3 T platform with efforts to maintain consistent procedures, the higher *b* value varied across scans (800–1000 s/mm^2^). Consequently, some degree of methodological variability in absolute ADC values cannot be excluded, and these values should be interpreted with caution. Third, the clinical course after diabetes onset was stable, and the diffusion-related imaging features and ADC values did not show clear time-dependent changes during follow-up. Therefore, the present data do not support the use of DWI/ADC to monitor disease activity, stratify risk, or guide management in clinical practice.

Our findings extend the existing literature by providing a long-term descriptive record of diffusion-related MRI features after the onset of ICI-induced diabetes. The main incremental value of this report is the 25-month documentation of these features beyond the acute phase. This long-term descriptive radiological course may serve as a reference for future prospective studies integrating serial imaging with endocrine and exocrine function testing, immune profiling, and, where feasible, histological correlation.

In conclusion, we documented long-term diffusion-related pancreatic MRI features and pancreatic atrophy in an individual with ICI-induced diabetes. Further prospective studies with appropriate comparators and tissue-based correlation are needed to clarify the biological substrate and clinical significance of these imaging findings.

## Data Availability

Data sharing is not applicable to this report as no data sets were generated or analyzed during the current study.
